# Enzymatic, Phyto-, and Physicochemical Evaluation of Apple Juice under High-Pressure Carbon Dioxide and Thermal Processing

**DOI:** 10.3390/foods9020243

**Published:** 2020-02-24

**Authors:** Ayesha Murtaza, Aamir Iqbal, Krystian Marszałek, Muhammad Amjed Iqbal, Shinawar Waseem Ali, Xiaoyun Xu, Siyi Pan, Wanfeng Hu

**Affiliations:** 1 College of Food Science and Technology, Huazhong Agricultural University, No.1, Shizishan Street, Hongshan District, Wuhan 430070, China; ayesha_murtaza2003@hotmail.com (A.M.); aamirraoiqbal@yahoo.com (A.I.); xiaoyunxu.88@gmail.com (X.X.); drpansiyi.hzau.edu@outlook.com (S.P.); 2 Key Laboratory of Environment Correlative Dietology, Huazhong Agricultural University, Ministry of Education, Wuhan 430070, China; 3 Department of Fruit and Vegetable Product Technology, Prof. Wacław Dąbrowski Institute of Agricultural and Food Biotechnology, 36 Rakowiecka, 02-532 Warsaw, Poland; krystian.marszalek@ibprs.pl; 4 Department of Chemistry and Food Toxicology, Institute of Food Technology and Nutrition, College of Natural Science, University of Rzeszow, Poland, Ćwiklińskiej 1A, 35-601 Rzeszow, Poland; 5 Institute of Agricultural and Resource Economics, University of Agriculture, Faisalabad 38000, Pakistan; amjadiqbal1775@yahoo.com; 6 Institute of Agricultural Sciences, University of the Punjab, Quaid-i-Azam Campus, Lahore 54590, Pakistan; shinawar.iags@pu.edu.pk

**Keywords:** polyphenols profile, polyphenol oxidase, peroxidase, thermal processing, high-pressure carbon dioxide

## Abstract

In this study, the changes in enzyme activities, total polyphenols, phenolic profile, and physicochemical properties from thermally (25–75 °C) and high-pressure carbon dioxide (HP-CO_2_) (25–65 °C/20 MPa)-treated apple juice were investigated. The HP-CO_2_ exhibited complete inactivation of polyphenol oxidase (PPO) at 65 °C, whereas PPO was still active at 75 °C under thermal processing (TP). Similarly, the relative activity of peroxidase (POD) significantly decreased by 71% at 65 °C under HP-CO_2_ processing, whereas TP was less effective. HP-CO_2_ and TP treatments at 65 °C reduced the browning degree (BD) value to 0.47 and 0.89, respectively. Thus, HP-CO_2_ inhibits the browning reactions caused by PPO and POD enzymes at each operating temperature. The concentration of epicatechin and catechin increased significantly with increasing temperature above 45 °C in TP-treated juices. HP-CO_2_ treatment increased the same phenolic compounds at 35 °C and 9 MPa, whereas high-temperature and -pressure conditions caused insignificant changes in concentration of epicatechin and catechin. Changes in others phenolic compounds were insignificant under TP and HP-CO_2_ treatment. Overall, HP-CO_2_ is a promising technology to get high-quality juices with lower enzyme activity.

## 1. Introduction

Apple is considered to be one of the most abundantly consumed fruits in the world. Apple juice is recognized as a health promoter because it contains a significant amount of nutrients and bioactive compounds such as polyphenols and organic acids [1,2]. Additionally, apple fruits showed the highest sources of phenolic compounds among daily consumed fruits [2,3]. Among apple polyphenols, flavanol monomers including catechin and epicatechin as well as procyanidin are most abundantly present and constitute more than 80% of total polyphenols in apples [4,5]. Phenolic compounds demonstrate important antioxidant properties and also take part in various health-promoting activities. Unfortunately, up to 90% of these compounds might be lost during clear juice processing [6]. For instance, nowadays, consumers prefer not-from-concentrate (NFC) juices rich in phenolic compounds and fiber. On the other hand, thermal processing (TP) also causes significant degradation of nutritional and sensorial values; therefore, scientists are still looking for new emerging techniques for food preservation that can maintain the food security and high nutritional value of food products.

Traditional juice processing also causes the enzymatic browning of juices, which is related to the high activity of oxidoreductive enzymes such as polyphenol oxidase (PPO) and peroxidase (POD), due to fast degradation of polyphenols to quinones, which further leads to the formation of browning pigments [2,3,7,8]. From this point of view, fast inhibition of browning reactions has gained much attention in the fruit and vegetable industry [3,9]. Thermal processing and addition of vitamin C has been conventionally used for the inactivation of these oxidoreductase enzymes, but according to European Union (EU) regulations, producers cannot give label declarations about 100% of juice, which might be an important drawback for premium-quality juices. Among all preservation techniques, thermal processing has significant drawbacks in terms of quality degradation, nutrient loss, flavor changes, and undesirable pigment formation during fruit and vegetables processing [3,5]. High-pressure carbon dioxide (HP-CO_2_) can be used as an alternative nonthermal technology for juice processing due to its high efficiency in enzyme inactivation and minimal effects on nutritional and sensory qualities of foods [2,10]. This method utilizes pressurized CO_2_ below 50 MPa for substantial enzyme inactivation at relatively mild operating conditions where thermal processing is not effective [11]. CO_2_ is a nonflammable, nontoxic, and inexpensive gas, which can be removed easily by out-gassing and depressurization during HP-CO_2_ processing. Thus, HP-CO_2_ is a cold pasteurization technique that utilizes CO_2_ to inactivate enzymes by pH lowering, cell membrane modification, cell disruption, and conformational changes in enzyme molecular structure [12,13].

The present study was conducted to investigate the influence of different temperatures and pressures on the enzyme activity, quality characteristics, and bioactive compounds in treated apple juice. For this purpose, a comparative study was also performed to compare the efficiency of both thermal and HP-CO_2_ treatments in term of enzymatic inactivation, browning inhibition, quality attributes, and phenolic composition in apple juice. Thus, the phenolic profile of HP-CO_2_-treated apple juice can be a fundamental theoretical guide for the commercialization of nonthermal technology.

## 2. Materials and Methods

### 2.1. Sample Preparation

Fresh apples (*Malus domestica*) at commercial maturity were bought from a native market in Wuhan, China. The fruits were washed, peeled, and cut into slices. After being sliced, a juice extractor was used to crush the apple slices. The juice was then filtered through a double cheese cloth to remove the impurities and coarse particles, and then the filtrate was centrifuged at 4000× *g* for 5 min (Eppendorf Centrifuge 5804, Eppendorf, Hamburg, Germany). The chemicals used in the experiments were of analytical grade or HPLC grade.

### 2.2. Processing Conditions

#### 2.2.1. HP-CO_2_ Processing Conditions

The mechanism of the HP-CO_2_ system was explained by Hu et al. [13], in which CO_2_ at purity of 99.5% obtained from Wuhan Co. (Wuhan, China) was allowed to enter inside the pressure vessel. The HP-CO_2_ processing was performed in a batch system apparatus containing two stainless-steel cylinders having an internal volume of 100 mL. Apple juice (30 mL) was put into a 50 mL sterile bottle for HP-CO_2_ processing (HA3000-30-type supercritical reaction unit, Jiangsu Nantong Huaan Co., Ltd., Nantong, Jiangsu, China). For each experiment, ethanol was used to sanitize the HP-CO_2_ vessel. After the desired temperatures of 25, 35, 45, 55, and 65 °C were achieved, the bottles containing juice samples were placed inside the chamber vessel, where the desired temperature and pressure for 20 min duration was given to the apple juice. The time required for pressurization and depressurization was not included in the treatment time. After HP-CO_2_ treatment, the vessel was gradually depressurized for 2–5 min, with a gradual decrease in juice temperature up to 8–12 °C. Then, the treated samples were rapidly cooled in a refrigerator for further analysis.

#### 2.2.2. Thermal Processing Conditions

Thermal processing of the apple juice was performed at different temperatures (25, 35, 45, 55, 65, and 75 °C) for 20 min in a water bath with a thermostat [11,14]. Apple juice (30 mL) was placed into test tubes, which were subjected to specified heat treatments in a water bath and then rapidly cooled in a refrigerator to stop thermal inactivation. Untreated juice was used as a control.

### 2.3. Browning Degree (BD) Analysis

The browning degree of untreated and treated apple juices was estimated by using a Multiskan FC spectrophotometer (Thermo Scientific, Waltham, MA, USA) according to Murtaza et al. [12]. The juice was centrifuged at 10,000× *g* (4 °C for 20 min) and the BD was determined by adding 100 μL of supernatant solution to the enzyme-linked immunosorbent assay (ELISA) plate and tested quickly at *λ* = 420 nm by using a simple kinetic method.

### 2.4. Color

The color of the apple juice was analyzed at an ambient temperature (20 ± 1 °C) with a chromometer (CR-400; Osaka, Japan). The L*, a*, and b* values of juice were measured, where L* indicates the treated sample brightness, L_o_ is the control sample “brightness”, a* indicates the greenness/redness of the treated sample, a_o_ is the redness/greenness of the control sample, b* indicates the yellowness of the treated sample, and b_o_ indicates the yellowness of control sample. The total color difference (ΔE) was determined by Equation (1):

(1) $$\Delta E={\left[{\left(L^{*}-L_{o}\right)}_{2}\;+\;{\left(a^{*}-a_{o}\right)}_{2}+{\left(b^{*}-b_{o}\right)}_{2}\right]}^{1/2}.$$

### 2.5. Physicochemical Analysis

The pH was estimated by using a digital Thermo Orion pH meter (Thermo Fisher Scientific Inc., Waltham, MA, USA). The total soluble solids (TSS) contents were determined by using a WAY-2S digital Abbe Refraction meter (Shanghai Precision and Scientific Instrument Co., Shanghai, China).

### 2.6. Enzyme Activity

#### 2.6.1. Polyphenol Oxidase (PPO)

Apple juice (2 mL) was mixed with 1% polyvinylpolypyrrolidone (PVPP) and 1% Triton X-100 to make a crude enzyme [12]. After 1 h of storage at 4 °C, juice was centrifuged at a high speed of 10,000 rpm for 20 min. The obtained crude enzyme was subjected to an oxidoreductase enzyme (polyphenol oxidase and peroxidase) activity assay. Each crude enzyme (50 μL) was individually mixed with 200 μL of substrate catechol solution (0.1 M) and 0.05 M phosphate buffer (pH 7.0). The absorbance of the extract was measured at 420 nm by a spectrophotometric method [15,16]. The PPO activity (Abs/min) was taken as the first linear part of the slope from the reaction curve [17]. The percentage of relative PPO activity was determined by using Equation (2):

(2) $$\mathrm{Relative}\;\mathrm{activity}\left(\mathrm{RA}\right)\;\left(\%\right)=\frac{\mathrm{Activity}\;\mathrm{of}\;\mathrm{juice}\;\mathrm{after}\;\mathrm{thermal}\;\mathrm{or}\;\mathrm{HP}-\mathrm{CO}2\;\mathrm{treatment}\;\left(A_{t}\right)}{\mathrm{Activity}\;\mathrm{of}\;\mathrm{untreated}\;\mathrm{juice}\;\left(A_{o}\right)}\;\times \;100$$

where A_o_ and A_t_ are the activity of the enzyme before and after HP-CO_2_ treatment, respectively.

#### 2.6.2. Peroxidase (POD)

POD activity was assayed by using methods described by Liu et al. [18] with slight modifications. An aliquot of the supernatant (100 μL) was added to 150 μL of mixture solution (200 mM phosphate buffer (pH 7.0), 5 mL guaiacol (0.5 M), and 10 mL hydrogen peroxide). As a control, 100 µL PBS was used, and the mixture solution absorbance was tested quickly using the simple kinetic method at a wavelength of 470 nm by a spectrophotometric method. The percentage of relative POD activity was determined as indicated by Equation (2).

### 2.7. Total Polyphenol Content (TPC)

TPCs were determined according to the Folin–Ciocalteu (FC) colorimetric method described previously by Dewanto et al. [19]. The optimally diluted supernatant (1.25 mL) was mixed with 1 mL of FC reagent (previously diluted 10-fold with distilled water), and after 6 min, the 10% Na_2_CO_3_ (1.8 mL) solution was added. The absorbance of the mixture was determined at 765 nm using a spectrophotometric method. Gallic acid as a standard was used, and the obtained results were expressed as milligrams of gallic acid equivalents (GAE) per liter of processed apple juice.

### 2.8. Phenolic Compounds Analysis

#### 2.8.1. Extraction and Purification of Apple Polyphenols

The HPLC analysis was performed according to the procedure established by [20,21], with some slight modifications. Briefly, 80 mL of juice was mixed at 25 °C with 400 mL of a 60% ethanol (*v*/*v*) solution for 4 h. Then, the juice was centrifuged for 15 min at 5000× *g*. The supernatant was evaporated under vacuum using a rotary evaporator at 45 °C and 30 MPa. The 20 mL solvent of 2% metaphosphoric acid and 20% ammonium sulfate, and ethyl acetate was used to extract phenolic compounds thrice. These extracts were combined and put in a rotary evaporator for drying at 45 °C and 30 MPa. The residue was dissolved in 5 mL of ethanol and then filtered through a filter membrane (0.22 μm) and stored at −20 °C.

#### 2.8.2. HPLC Polyphenol Profile Analysis

The phenolic compounds were analyzed by HPLC (Waters 2695; C18 column (250 × 4.6 mm × 5 μm size particle) and the absorbance was measured at 280 nm by a Waters 2478 Dual λ Absorbance Detector. The mobile phase of acidified water containing 1% formic acid (A) and acetonitrile (B) was used. The setting procedure of the gradient was as follows: 0 min, 20% B; 17 min, 21.5% B; 17.5 min, 68% B; 40 min, 68.3% B; 41 min, 100% B; 51 min, 20% B, and held for 2 min. The volume of injection was 20 μL and the flow rate was 1.0 mL/min. All HPLC-grade standards (ferulic acid, epicatechin, catechins, phloridzin, phloretin, caffeic acid, rutin, and chlorogenic acid) were purchased from Sigma Chemicals. Identification of each peak was performed on the basis of comparing their retention times with their known standards. The concentration of phenolic contents was expressed as milligrams of each compound per liter of juice.

### 2.9. Statistical Analysis

Analysis of variance (ANOVA) was adopted in data Analysis. The results are presented as the mean ± standard deviation of three replicates, performed on a number of samples for each experiment. Microsoft Origin Pro 9.0 (Origin Lab, Northampton, UK) was used for analysis, and the comparison of means was made by Tukey’s test. The mean was taken at the 95% level of significance.

## 3. Results and Discussion

### 3.1. Effect of Thermal and HP-CO_2_ Treatment on Color Changes, pH, and TSS

The effects of thermal and HP-CO_2_ treatments on the physicochemical characteristics of apple juice under different conditions of pressure and temperature are presented in Table 1. The pH and TSS contents in untreated juices were 3.67 and 12.45, respectively. HP-CO_2_-treated juice showed a greater decline in pH compared with thermal-treated juice, which might have been due to the dissolved CO_2_ gas, distributing the carbonates and bicarbonates in the juice, thereby increasing the acidity and lowering the pH value of the juice. This finding was similar to our previous studies on carrot, quince, and apple juices, where pH was reduced under HP-CO_2_ treatment [11,20,22].

Color is an important parameter to judge the quality of juice because it has a great influence on the overall acceptability of juice. The total color difference (∆E) of HP-CO_2_- and thermally treated juice is shown in Table 1. Color changes could be due to the high susceptibility of juice to browning reactions because of the presence of active oxidizing enzymes [23]. An increase in L value (brightness) was observed in HP-CO_2_-treated juice compared with thermal-treated juice. The ∆E of thermally treated juice was significantly lower compared with HP-CO_2_-treated juice. The highest ∆E (14.54) was observed at 65 °C under HP-CO_2_ treatment, which was higher than the thermal-treated juice at 65 °C (8.12). The ∆E values for all treatments were greater than 2, indicating that both thermal and HP-CO_2_ treatments led to visible color changes in apple juice [24]. A similar trend was observed by Marszałek et al. [2], when they observed the quality changes of apple juice under super critical carbon dioxide (SCCD) treatments. These authors found a highly visible change of yellow color of untreated juice into brown after SCCD treatment, attributing these changes to active oxidizing enzymes. This color change might be due to the browning reactions of oxidoreductase enzymes such as PPO and POD, which could lead to the quality degradation of juice.

### 3.2. Effect of Thermal and HP-CO_2_ Treatment on BD

**Figure 1:**
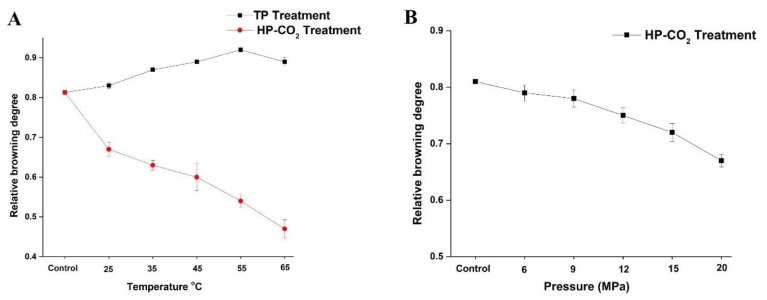
Changes in browning degree (BD) value of apple juice under thermal processing (TP) (25–75 °C) and High pressure carbon dioxide (HP-CO_2_) (25–65 °C for 20 MPa) for 20 min (**A**) and HP-CO_2_ treatment at different pressures (6–20 MPa, 30 °C) for 20 min (**B**). Data presented as the mean ± SD (standard deviation).

The effect of thermal and HP-CO_2_ treatments at different temperatures and pressures on the BD of apple juice is shown in Figure 1. Browning in apple juice is considered to be induced by oxidoreductive enzymes. PPO and POD are closely related to the enzymatic browning of fruit products. The untreated juice showed a browning degree value of 0.81, which increased to 0.83 and 0.87 by increasing the temperature to 25 and 35 °C after TP. The BD increased with the increasing temperature and reached up to 0.92 at 55 °C, but a slight reduction in the BD (0.89) value occurred at 65 °C. Thus, a higher temperature under TP could not effectively inhibit the browning reactions caused by enzyme activity. According to the analysis in Figure 1A, the increase of temperature led to the increase in polyphenol concentration, stimulating the enzymatic activity of PPO by increasing the relative browning value of juice.

HP-CO_2_ treatment at a high temperature caused the inhibition of enzyme activity, resulting in the reduction of the browning degree after HP-CO_2_ treatment of juice [11]. Compared with control and TP juice, HP-CO_2_ treatment significantly reduced the BD value to 0.63 and 0.60 at 35 and 45 °C, respectively, and to below 0.47 when a high temperature of 65 °C was applied. This phenomenon may be justified by the higher inactivation of native enzymes by CO_2_ at high pressure causing structural changes in enzymes. Furthermore, CO_2_ density decreased by increasing the temperature under HP-CO_2_ treatment, which improved the diffusivity of CO_2_, accelerating the molecular collisions of CO_2_ with enzymes, ultimately reducing the enzymatic activity and enzymatic browning in the juice [10].

Figure 1B shows the influence of HP-CO_2_ treatment at different pressures (6, 9, 12, 15, and 20 MPa) on the browning degree of apple juice. The browning degree gradually reduced by increasing the pressure. The BD value at 6 MPa was 0.79 and this value declined to 0.78 and 0.72 when the pressure increased to 9 and 15 MPa, respectively. The reduction in browning degree at high pressure may be due to the more inhibition of enzymes under high pressure of CO_2_ gas during HP-CO_2_ treatment or by slowing down non-enzymatic browning reactions in a lower pH environment. Thus, the increase in CO_2_ pressure is directly related to the PPO inactivation and browning inhibition under the HP-CO_2_ treatment. High pressure connected with CO_2_ may have inhibited the browning reaction caused by PPO at each operating temperature. PPO relative activity was lower for HP-CO_2_ treatment compared with TP, proving that HP-CO_2_ treatment was more effective at enzyme inhibition [10].

The PPO enzyme in Fuji apple juice was completely inactivated by HP-CO_2_ treatment at 22 MPa (60 °C) for 10 min [25]. The BD value of HP-CO_2_-treated quince juice was significantly lowered in comparison with thermal treatment [11]. BD is considered to be closely related to PPO enzyme actions; thus, HP-CO_2_ treatment can effectively inactivate the enzyme as compared to TP for the prevention of browning in apple juice.

### 3.3. Effect of Thermal and HP-CO_2_ Treatment on PPO Inactivation

**Figure 2:**
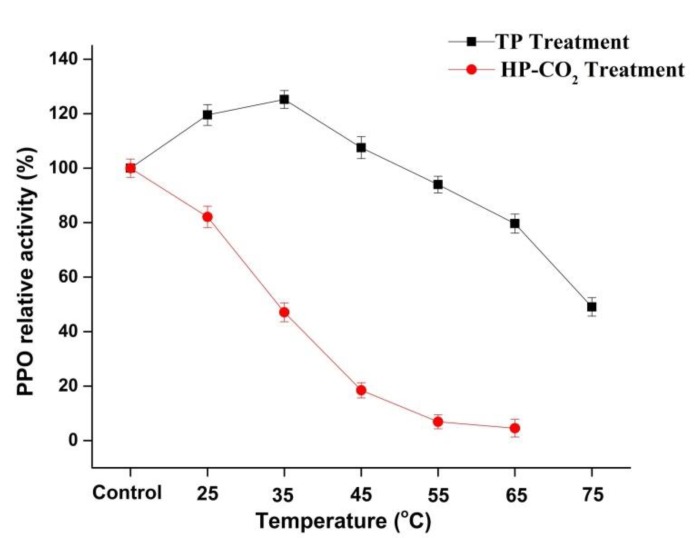
Relative activity of polyphenol oxidase (PPO) in apple juice under thermal processing (TP) (25–75 °C) and high pressure carbon dioxide (HP-CO_2_) (25–65 °C for 20 MPa) treatments for 20 min. Data presented as the mean ± SD (standard deviation).

The relative activity (RA) of apple PPO during the thermal and HP-CO_2_ (20 MPa) treatments at a temperature range from 25 to 65 °C for 20 min is shown in Figure 2. The highest PPO relative activities of 119.54% and 125.30% were observed at 25 and 35 °C, respectively. PPO in apple juice after TP showed an increase in relative activity by increasing temperature, but a temperature higher than 35 °C caused a lowering of the relative activity of PPO with increasing temperature. In plants, PPO exists in many (immature, mature, active, and latent forms) isoforms and the increased activity of PPO in apple juice after TP might be due to the activation of latent PPO [26,27]. Many fruits and vegetables, including iceberg lettuce, apples, grapes, and peaches, have latent PPOs [28]. There are a number of studies in which the increase in plant PPO activity found following thermal treatment could have been due to the activation of a latent PPO precursor in the enzyme extracts [28,29]. Furthermore, in our study, PPO relative activity decreased to 97.74% and 39.07% at 65 and 75 °C, respectively. However, results of HP-CO_2_-treated apple juice showed that the RA of PPO decreased to 82.09%, 47.07%, and 18.45% with rising temperatures from 25, 35, and up to 45 °C, respectively, but caused total inactivation at 55 and 65 °C (Figure 2).

HP-CO_2_-treated juice had significantly (*p* ≤ 0.05) lower PPO activity compared with thermal-treated juice at the same temperatures. For inactivation of enzymes, TP has been used with negative effects on the quality of foods [2,3,30]. Many researchers have found that PPO inactivation under HP-CO_2_ treatment increased with increasing temperature, pressure, and treatment time [18,31]. This enzymatic inhibition during HP-CO_2_ might have been due to the structural changes in PPO, resulting from the pH-lowering effects after CO_2_ dissolved in solution [11]. Li et al. [32] reported that PPO activity in apple under HP-CO_2_ treatment showed a biphasic change of initial activation and sudden inactivation at 35–55 °C for 15 min (20 MPa). The higher activity of PPO could lead to an increase in browning due to the oxidation reactions of polyphenol. The loss in PPO activity might be due to the modification of the enzyme structure induced by HP-CO_2_ treatment [11]. Thus, it was found that HP-CO_2_ performed more effectively at enzymatic inactivation compared with thermal treatment.

### 3.4. Effect of Thermal and HP-CO_2_ Treatment on POD Inactivation

**Figure 3:**
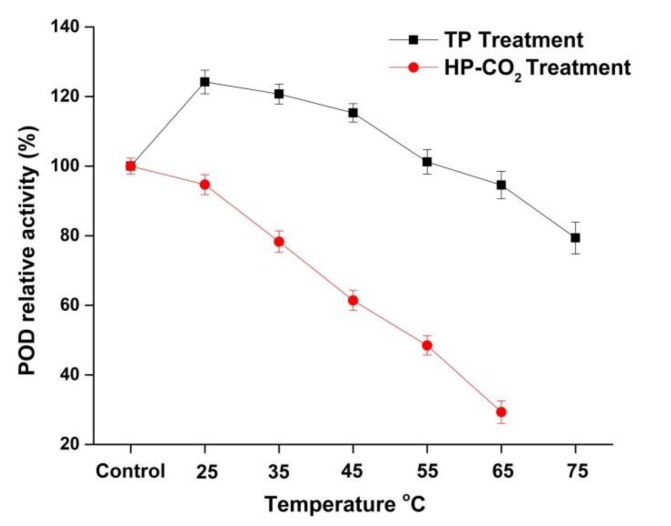
Relative activity of peroxidase (POD) in apple juice under thermal processing (TP) (25–75 °C) and high pressure carbon dioxide (HP-CO_2_) (25–65 °C for 20 MPa) treatment for 20 min. Data presented as the mean ± SD (standard deviation).

The RA of POD in apple juice after thermal and HP-CO_2_ treatments is shown in Figure 3. The relative activity of POD in TP juice was 124.19% and 120.72% at 25 and 35 °C, respectively. The relative activity of POD in TP apple juice was 115.31%, 94.56%, and 79.35% at 45, 65, and 75 °C, respectively, indicating that POD was more stable compared with the PPO enzyme in this study. As shown in Figure 3, HP-CO_2_-treated juice indicated a higher decrease in POD activity: 94.68%, 78.28%, and 61.39%, respectively, for 25, 35, and 45 °C in comparison with TP. These findings prove that the higher temperature under HP-CO_2_ caused a more rapid inactivation of POD. Our research also confirmed that different enzymes have different stability at the same process parameters. HP-CO_2_ treatment at 20 MPa caused complete inactivation of PPO at 55 and 65 °C, whereas POD showed activity as high as 48.44% and 29.32% at the same temperatures and pressure. Similarly, the POD activity in watermelon juice was reduced to 82.9% at 95 °C, while HP-CO_2_ treatment at 20 MPa and 50 °C for 20 min caused a 75% reduction in POD activity [18]. It is believed that browning is generally due to PPO- and POD-mediated oxidation of phenols [33]. POD is considered to be a more heat-stable enzyme than PPO and it may show important activity up to 75 °C [34]. This might be due to the structural differences of PPO and POD [35], as POD is considered to be a larger molecule with a complex structure; thus, longer heat durations are needed to inactivate the complex structure of POD [34]. Furthermore, PPO and POD inactivation under HP-CO_2_ treatment could be due to the changes in secondary and tertiary structures [25,36]. It can be concluded that the POD enzyme was more pressure and thermal resistant than PPO. The different trend in the resistance of PPO and POD could be due to the variable capacity of expansion in their structures during thermal and pressure application. Overall, HP-CO_2_ caused more enzyme inactivation due to the decrease in pH value and more structural modifications induced by high pressure.

### 3.5. Effect of Thermal and HP-CO_2_ Treatment on Total Phenolic Components

**Figure 4:**
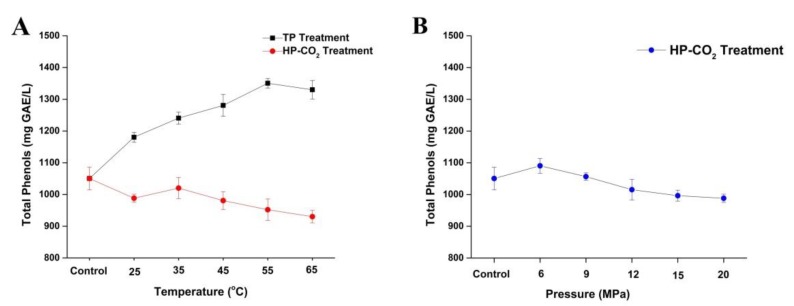
Changes of total polyphenol content (TPC) in apple juice under thermal (25–65 °C) and HP-CO_2_ (25–65 °C for 20 MPa) treatment for 20 min (**A**) and HP-CO_2_ treatment at different pressures (6–20 MPa, 30 °C) for 20 min (**B**). Data presented as the mean ± SD (standard deviation). GAE stands for Gallic acid equivalent.

Total phenolic content in apple juice after thermal and HP-CO_2_ treatment is shown in Figure 4. The concentration of total phenolic content in untreated juice was 1050.45 mg GAE/L, which is consistent with the previous studies of apple juices, where phenolic values ranging between 100 and 3000 mg/L were attained [37,38,39]. Total phenol contents slightly increased to 1180.27 mg GAE/L at 25 °C under thermal treatment, which was higher than the HP-CO_2_-treated juice (988.31 mg GAE/L) at the same temperature. Thermal treatment gradually increased the total phenolic contents by increasing the treatment temperature from 25 to 55 °C (Figure 4A). This may be due to the reason that phenolic compounds in apple juice are mostly bounded with polysaccharides, which can be released by thermal treatment of juice as well as according to the higher concentration of Maillard reaction products, which also have high affinity to the FC reagent. A similar finding was found by Huang et al. [40], in which the phenolic content in apricot nectar increased after thermal processing.

Apple juice after HP-CO_2_ treatment generally exhibited decreased phenolic contents by increasing temperature under 20 MPa (Figure 4A). Compared with untreated juice, the phenolic content decreased to 988.31 mg GAE/L at 25 °C, while 1020.12 mg GAE/L of phenolic compounds was observed at 35 °C. This might be due to the effect of higher temperatures, which could release the bounded polyphenol. In our study (except at 35 °C), HP-CO_2_ treatment showed slight reduction of phenolic contents by increasing treatment temperature up to 952.23 and 930.17 mg GAE/L of phenolic contents noted in samples treated at 55–65 °C. This can be attributed to the fact that HP-CO_2_ possibly contributed to the hydrolysis of polyphenols under the lower acidity of the environment; thus, high CO_2_ pressure could stimulate the release of simple phenolic compounds from the more complex structures (higher polyphenols) [41].

For a better understanding of the degradation of phenolic contents, the effect of different pressure treatments from 6 to 20 MPa is shown on Figure 4B, where the highest decrease of total polyphenol contents was observed at the high pressure of 20 MPa. The degradation mechanism of phenolic compounds after HP-CO_2_ is very complicated and unknown. The dissolved CO_2_ gas inside the juice could induce acidic conditions by generating high reactive species such as superoxide (O_2_-), hydroxyl radical (OH.), and hydrogen peroxide (H_2_O_2_), which are highly reactive with phenolic compounds [42]. Overall, HP-CO_2_ treatment at different temperatures and pressures may lead to the degradation of phenolic compounds. Many researchers found significant modifications in total polyphenolic content in juice after HP-CO_2_ treatment, leading to the degradation of phenolic compounds by increasing pressures [2]. Moreover, PPO activity can also be inhibited by applying high temperatures and pressures in juice, thus facilitating the PPO reaction with phenolic compounds, as a result caused by phenolic compound degradation [31]. Additionally, several polyphenols can be masked by polymeric phenolic compounds [43,44]. Therefore, it can be predicted that acidic hydrolysis can happen under an HP-CO_2_ environment.

### 3.6. Phenolic Profile of Thermally and HP-CO_2_-Treated Apple Juice

**Figure 5:**
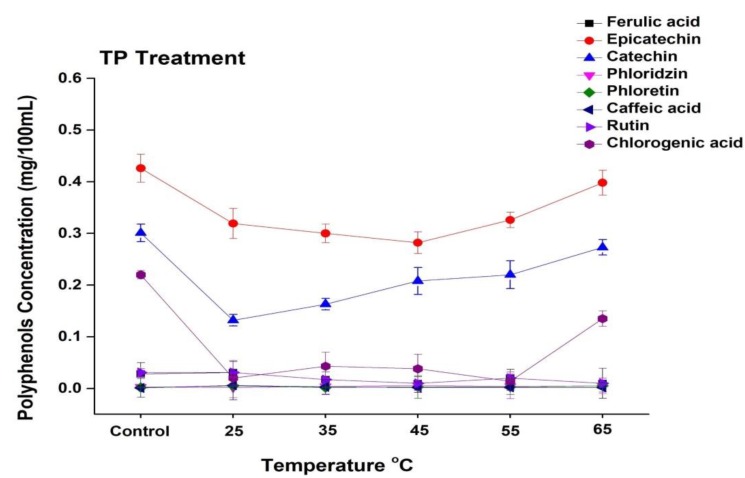
Changes in the phenolic profile of apple juice subjected to thermal treatment (25–65 °C) for 20 min. Data presented as the mean ± SD (standard deviation).

**Figure 6:**
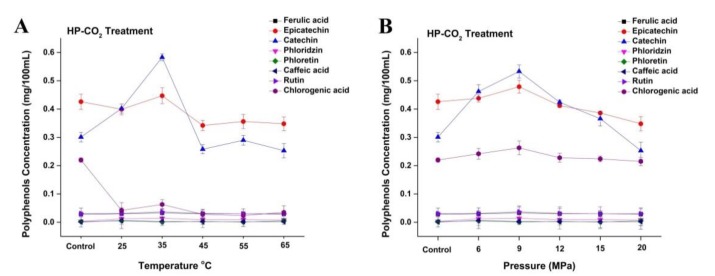
Changes in phenolic profile of apple juice subjected to different HP-CO_2_ temperatures (25–65 °C for 20 MPa) for 20 min (**A**) and different HP-CO_2_ pressures (6–20 MPa at 30 °C) for 20 min (**B**). Data presented as the mean ± SD (standard deviation).

In general, apple juice contains a variety of phenolic compounds, including chlorogenic acid, epicatechin, catechin, rutin, caffeic acid, phloretin, and chlorogenic acid. The current study revealed that the concentrations of epicatechin, catechin, and chlorogenic acid in apple juice are higher, while ferulic acid, rhizoctin, caffeic acid, and rutin are present in lower concentrations (Figure 5 and Figure 6). The phenolic profile was significantly changed during the processing of apple juice. The effect of TP under the temperature range from 25 to 65 °C on selected phenolic compound concentrations is shown in Figure 5. The concentration of epicatechin in untreated juice was 0.42 mg/100 mL, which decreased up to 0.31 mg/100 mL at 25 °C and increased up to 0.39 mg/mL at 65 °C. The concentration of catechin and chlorogenic acid increased at high treatment temperature. Compared with the 0.13 mg/100 mL noted for 25 °C thermally treated sample, the catechin concentration increased to 0.27 mg/100 mL at 65 °C. Thus, thermally treated juice at a high temperature (65 °C) showed higher concentrations of polyphenols. Similar results were found by Huang et al. [40], who reported that a high temperature (110 °C, 8.6 s) significantly increased four individual phenolic compounds in apricot nectar, including (-)-epicatechin and (+)-catechin. Kim et al. [45] reported that epicatechin and procyanidin B2 are the major reactants in the enzymatic browning of apple. Hence, thermal treatment enhanced the enzymatic activity and browning degree in apple juice, which might have been due to the enhanced concentration of different polyphenols such as epicatechin, catechin, and chlorogenic acid at high temperatures.

The concentration of polyphenols such as catechin and epicatechin initially increased to 0.58 and 0.44 mg/100 mL, respectively, at 35 °C under HP-CO_2_ (20 MPa) treatment (Figure 6A). These polyphenol contents showed a decline at the relatively higher temperature of 45 °C and had lower concentrations of catechin and epicatechin (0.25 and 0.34 mg/100 mL, respectively). Several studies have revealed that phenolic compounds during product processing are greatly reduced during the physical application of temperature and pressure [4]. These changes could be caused by the hydrolysis of polyphenols due to the increased acidity during pressurization, releasing polyphenolic compounds and enzymes that subsequently react to form of polymeric brown pigments [2]. The concentration of phenols such as epicatechin and catechin initially increased to 0.47 and 0.53 mg/100 mL, respectively, by increasing the pressure from 6 to 9 MPa under a constant temperature of 30 °C (Figure 6B), whereas the concentration of chlorogenic acid slightly increased to 0.26 mg/100 mL at 9 MPa. However, the concentration of polyphenols decreased by increasing pressure after 9 MPa. Similarly, HP-CO_2_ treatment of mulberry juice at 15 MPa (25 °C) for 10 min reduces the total phenolic contents as compared with untreated mulberry juice [24]. The decrease in polyphenol contents under HP-CO_2_ might be due to reason that high pressure could degrade the polyphenol structure and could make a pressure gradient between the inside and outside of the structure, which causes the quick discharge of CO_2_ gas containing polyphenol, resulting in the loss of polyphenol contents [22].

## 4. Conclusions

HP-CO_2_ processing was more effective at lowering the enzyme activities and phenolic contents of apple juice in comparison with thermally processed juice. The enzymes (PPO and POD) in apple juice retained most of their relative activities even at high temperatures of 65–75 °C under thermal processing, while HP-CO_2_ treatment showed complete inactivation of these enzymes at 55 and 65 °C. Thermal processing resulted in a high level of the BD value, while HP-CO_2_ treatment appeared to inhibit the browning reaction, which seems to be an important advantage of this technique compared with thermal treatment. Total phenolic contents increased with increasing temperature under TP, while HP-CO_2_ slightly reduced the total phenolic content, indicating the stability of phenolic compounds during HP-CO_2_ treatment. The significant degradation of individual polyphenols (catechins 0.25 and epicatechin 0.35 mg/mL) was noted at 45 °C under HP-CO_2_ and also showed a gradual decline during the increase in pressure under HP-CO_2_ treatment. Moreover, it can be concluded that HP-CO_2_ treatment can be a useful nonthermal approach to enhance the nutritional and physicochemical quality of apple juice. A high level of enzyme inactivation is essential in preserving the quality of food, especially fruit and vegetable products; therefore, further studies need to be undertaken to improve the efficiency of the HP-CO_2_ process in this aspect.

## Figures and Tables

**Table 1:** pH and brix of apple juice from thermal and high-pressure carbon dioxide (HP-CO_2_) treatments.

Treatment	Temperatures	pH	∆E	Brix
**Control (Untreated)**		3.67 ± 0.01 ^a,b^	-	12.45 ± 0.21 ^a^
**Thermal-treated (20 min)**	25	3.68 ± 0.07 ^a^	6.30 ± 0.68 ^e,f^	12.34 ± 0.15 ^a^
	35	3.60 ± 0.01 ^a,b^	6.97 ± 1.23 ^e,f^	12.46 ± 0.19 ^a^
	45	3.60 ± 0.03 ^a,b^	7.10 ± 0.51 ^e,f^	12.48 ± 0.22 ^a^
	55	3.59 ± 0.11 ^a,b^	7.45 ± 0.84 ^d^	12.41 ± 0.16 ^a^
	65	3.60 ± 0.13 ^a,b^	8.12 ± 0.45 ^c,d^	12.43 ± 0.17 ^a^
**HP-CO_2_-treated (20 MPa, 20 min)**	25	3.58 ± 0.03 ^a,b^	7.20 ± 0.31 ^d^	12.37 ± 0.27 ^a^
	35	3.50 ± 0.13 ^b^	9.09 ± 0.75 ^c^	12.35 ± 0.06 ^a^
	45	3.55 ± 0.04 ^b^	10.85 ± 0.59 ^b,c^	12.15 ± 0.05 ^a^
	55	3.35± 0.08 ^c^	12.32 ± 0.90 ^b^	11.95 ± 0.07 ^a^
	65	3.23 ± 0.02 ^d^	14.54 ± 0.45 ^a^	11.82 ± 0.02 ^a^

Data presented as the mean ± SD (standard deviation). Different letters represent the significant difference among means (*p* ≤ 0.05).
